# AGE-FRIENDLY UNIVERSITY IN PRACTICE: DEVELOPING AN AGE INCLUSIVITY MICRO-CREDENTIAL PROGRAM

**DOI:** 10.1093/geroni/igae098.1684

**Published:** 2024-12-31

**Authors:** Serena Hasworth

**Affiliations:** Portland State University, Portland, Oregon, United States

## Abstract

This session will highlight findings from PSU’s “Age-Friendly University Campus Report” (Silverstein et al., 2021) and provide an overview of the activities to improve age-friendliness through the Better with Age Initiative, a program that invests in students pursuing career pathways in aging and faculty conducting aging-related research. Presenters will describe efforts to develop a free, self-paced Age Inclusivity micro-credential program for students and faculty at PSU in which participants learn to describe local and national growth of aging populations, analyze the intersectional experience of aging, reflect on personal attitudes of aging, determine how older adults play a role in society at large, and explore career opportunities in the aging field. Micro-credentials provide tailored learning experiences for students, faculty, and staff that supplement and enrich current curricular and professional development offerings. Presenters will describe how these programs have the potential to reach students who may not be interested in pursuing a formal degree or minor focused on aging, but who want to set themselves apart on the job market by demonstrating their knowledge and skills related to serving older adults in their unique field.

